# Can We Count on Global Health Estimates?

**DOI:** 10.1371/journal.pmed.1001002

**Published:** 2010-11-30

**Authors:** The PLoS Medicine Editors

**Related Articles**

1. Byass P (2010) The Imperfect World of Global Health Estimates. PLoS Med 7: e1006. doi:10.1371/journal.pmed.1001006
2. Boerma T, Mathers C, Abouzahr C (2010) WHO and Global Health Monitoring: The Way Forward. PLoS Med 7: e373. doi:10.1371/journal.pmed.1000373
3. Murray C, Lopez A (2010) Production and Analysis of Health Indicators: The Role of Academia. PLoS Med 7: e1004. doi:10.1371/journal.pmed.1001004
4. Sankoh O (2010) Global Health Estimates: Stronger Collaboration Needed With Low- and Middle-Income Countries. PLoS Med 7: e1005. doi:10.1371/journal.pmed.1001005
5. Graham W, Adjei S (2010) A Call for Responsible Estimation of Global Health. PLoS Med 7: 1003. doi:10.1371/journal.pmed.1001003

This week *PLoS Medicine* publishes a cluster of articles discussing the current state of global health estimates and debating the way into the future [1]–[5].

Estimates of global health indicators—which give insight into death and disease rates, document advances in health and development, and help policymakers monitor progress—are a necessary evil. They are absolutely essential to improving global health, but they are always unsatisfyingly imperfect. Estimates are estimates—that is, they are not true measurements of health and death. They rely on often inadequate data to create a best guess. Some estimates are undoubtedly better than others, but even with advanced statistical techniques and complex modeling tools it is often frustratingly difficult to interpret and judge the estimates that result and to have complete confidence in their accuracy.

As such, estimates are often debated, sometimes fiercely. The idea for a cluster of articles on this topic came from Ties Boerma and Colin Mathers at WHO, who submitted an article to *PLoS Medicine* laying out their reflections on WHO's estimate work following the high-profile publication of maternal and child mortality estimates by an academic group in advance of the UN's own release of estimates. We felt that a range of viewpoints on the burning issues in health indicator estimates, and on the future of the field, would serve readers best, so we commissioned a group of articles to accompany the piece by Boerma and colleagues [2].

That academic institutions are now in the game of estimate-making, introducing competition in an area that was once the dominion of UN agencies, provides some impetus for the cluster. But the fact that so much has been made of the differences between different estimates is another driver. On the one hand, why does it matter that either 380,000 [6] or 500,000 [7] women die every year trying to give birth—these are both astonishing and deplorable numbers. On the other hand, that national authorities and policymakers working for decades with one set of (UN) numbers might be blind-sided by new, “improved” estimates tracking their country's health and development [8],[9] means something important is lost in translation and must be explored.

We commissioned articles from several experts to provide insights and opinion on what the estimates mean for global health, how their generation can be improved, and how to move forward with better data, measurement, and coordination. Representing very different institutional and political orientations, the experts nevertheless agree that the debate about health estimates highlights the relative importance of “the global” and “the local.” For example, each commentator emphasizes the importance of improving the quantity and quality of individual health data and of improving the role of local experts at the country level. This suggests that contentiousness about health indicator estimates operates too much at the level of the global and political, and not enough at levels where real data are generated and interpreted.

Medical journals would serve the field best by equally considering original research of both country and global data estimates (following the quality of the science rather than any specific policy agenda), and by publishing incisive commentary and analysis on how these estimates are shaped, fueled, and improved. Since at the very core of the debate about health estimates is the issue of quality, availability, and transparency of data, medical journals could also continue to advocate for data sharing (as PLoS often has), and to support initiatives that call for action on health data—such as the H8 position paper from the eight leading global health agencies published in *PLoS Medicine* earlier this year [10]. The last thing the field needs is yet more divisions.
